# Nebivolol treatment improves hypertension-induced endothelial cell dysfunction by reducing TGF-β1-dependent senescence and normalizing mitochondrial indices

**DOI:** 10.1097/HJH.0000000000004391

**Published:** 2026-07-29

**Authors:** Paweł Uruski, Justyna Mikuła-Pietrasik, Andrzej Tykarski, Krzysztof Książek

**Affiliations:** a Poznan University of Medical Sciences, Department of Hypertension, Angiology, and Internal Medicine; b Poznan University of Medical Sciences, Department of Pathophysiology of Ageing and Civilization Diseases; c Prince Mieszko I, Poznan Medical University of Applied Sciences, Faculty of Medical Sciences, Poznan, Poland

**Keywords:** antihypertensive therapy, endothelial cells, hypertension, nebivolol

## Abstract

**Objectives:**

Serum from patients with hypertension (HT) causes endothelial cell (EC) damage, leading to senescence and dysfunctional phenotype. This study investigated whether serum from patients treated with the antihypertensive drugs could normalize EC activity.

**Methods:**

This study involved 71 patients with newly diagnosed HT, who were randomly assigned to one of three groups based on the antihypertensive treatment: amlodipine, nebivolol, or perindopril. Serum samples collected before and 6 weeks after treatment were applied to ECs in vitro to assess their angiogenic activity, cellular senescence, mitochondrial metabolism, and oxidative stress.

**Results:**

Results showed that exposure of ECs to serum from patients treated for 6 weeks significantly altered EC function, with varying effects among the drugs. Serum from nebivolol-treated patients produced the most consistent benefits, reducing EC proliferation and HIF-1α expression, likely due to lower levels of angiogenic factors such as angiopoietin-1, basic fibroblast growth factor (bFGF), insulin-like growth factor 1 (IGF-1), and vascular endothelial growth factor (VEGF). Additionally, this serum contained reduced levels of pro-inflammatory cytokines (E-selectin, P-selectin, monocyte chemoattractant protein-1 (MCP-1), and tumor necrosis factor α (TNFα)) and lower TGF-β1, which are linked to HT-related EC senescence. Nebivolol treatment decreased senescence biomarkers such as SA-β-Gal, 53BP1, and p16, with SA-β-Gal reduction comparable to that of TGF-β1 neutralizing antibodies. Oxidative stress was reduced, indicated by lower oxidized DNA product levels.

**Conclusions:**

Nebivolol was the most effective at reducing the factors, associated with HT induced cellular senescence of endothelium, through reducing TGF-β1.

## INTRODUCTION

Hypertension (HT) is a persistent condition in which the force of blood against arterial walls is consistently elevated. This condition, if not adequately treated, can lead to several health issues, including atherosclerosis, heart disease, stroke, and kidney damage [1].

Endothelial cells (ECs) play a crucial role in the progression and outcomes of HT. Their dysfunction, which stems from oscillatory blood flow, inflammation, and the overproduction of reactive oxygen species (ROS), contributes to HT development. Mechanistically, it is associated with decreased expression of endothelial nitric oxide synthase (eNOS), reduced nitric oxide (NO) production, increased release of vasoconstrictive agents and mitogens for smooth muscle cells, and the activation of the renin–angiotensin system (RAS). These events collectively lead to increased peripheral vascular resistance, vessel wall remodeling, and stiffness, which elicit a myogenic response and an increase in pulse pressure [2,3]. A specific vicious cycle can perpetuate the progression of HT, where elevated blood pressure (BP) leads to further deterioration of ECs. Mechanical shear stress damages cells and triggers oxidative stress, along with local inflammatory responses. As a result, leukocytes, particularly macrophages, are recruited, leading to an even greater release of ROS and pro-inflammatory cytokines [2,4].

Several classes of medications are used to treat elevated BP. The most widely used include thiazide diuretics, β-blockers, angiotensin-converting enzyme inhibitors (ACEi), calcium channel blockers (CCBs), and aldosterone antagonists. These drugs can be used individually or combined in a multidrug strategy [5]. While the primary goal of these drugs is to reduce BP, they also impact ECs. For instance, medications targeting the RAS, such as ACEi and angiotensin receptor blockers (ARBs), stabilize EC function by activating the angiotensin II type 2 receptor (AT2R) [6]. This activation leads to increased NO production, vasodilation, and exerts antiproliferative and anti-inflammatory effects [7]. Despite these findings, studies investigating the effects of various antihypertensive strategies on EC activity remain limited and do not fully explore all aspects of their dysfunction.

We have recently observed that ECs can be affected by serum from patients with HT. Notably, the composition of HT serum differs from that of healthy individuals and alters the secretory and genetic profiles of ECs, leading them to adopt cancer-promoting characteristics [8]. Additionally, HT serum accelerates EC senescence [9], a process that may hypothetically contribute to their dysfunctional phenotype [10].

Taking these findings into account, we aimed to examine whether antihypertensive treatment could alter the serum characteristics associated with HT and thus mitigate its pro-senescence activity. To this end, we designed a project involving patients with newly diagnosed HT who were treated with different antihypertensive medications. Serum samples collected before and after treatment were applied to ECs in vitro to assess their angiogenic activity, cellular senescence, mitochondrial metabolism, and oxidative stress.

## MATERIALS AND METHODS

### Chemicals and consumables

Unless otherwise stated, all chemicals and plastic disposables were obtained from Sigma (St. Louis, MO). A specific TGF-β1 neutralizing antibody and an isotype control IgG were obtained from R&D Systems (Abingdon, UK).

### Hypertensive patient characteristics

This study involved 71 patients who were newly diagnosed with primary hypertension. Hypertension was defined according to the 2018 Guidelines of the European Society of Cardiology [11], which stipulate that HT is present when systolic blood pressure (SBP) is ≥ 140 mmHg and/or diastolic blood pressure (DBP) is ≥90 mmHg. Participants were excluded if they showed signs of secondary HT, had received prior hypotensive treatment, or had chronic kidney disease with an estimated glomerular filtration rate (eGFR) of <60 ml/min/1.73 m^2^. Additionally, individuals who were underage, pregnant, or breastfeeding were also excluded. The study received approval from the institutional bioethics committee (Poznan University of Medical Sciences Bioethics Committee, consent number 415/17, August 6, 2017), and all participants provided written informed consent. The study was conducted in accordance with the principles of the Declaration of Helsinki. Serum samples were de-identified prior to laboratory analyses. Each patient underwent a comprehensive medical interview and examination to identify potential causes of secondary HT and to review current medications. Office blood pressure was measured using the Omron 705 IT device, with the average of three consecutive readings recorded while participants were seated. Blood samples were collected from the HT patients at the time of diagnosis, designated as time-point 0 (T0). Following this, patients were randomly assigned to one of three groups based on the antihypertensive treatment they received, which included amlodipine (5 mg), nebivolol (5 mg), or perindopril (5 mg), with the allocation being stratified by age and gender to ensure a balanced sample. A second blood sample was taken six weeks after treatment initiation (designated as time-point 1; T1). Serum samples were immediately centrifuged and stored in aliquots at -80°C until required. The characteristics of HT patients are detailed in Table 1. Blood samples were obtained from all patients in a fasted state. Serum samples were applied individually; thus, each patient's serum represents one biological replicate (*N* = number of patients per arm). Samples were aliquoted to minimize freeze–thaw; each aliquot was thawed once for experiments. For cell-based assays, measurements were performed in technical replicates and averaged per patient serum prior to group-level statistics.

**TABLE 1:** Characteristics of patients whose serum samples were used in the study

Parameter	HT patients			
	Amlodipine	Nebivolol	Perindopril	*P*
*n*	23	24	24	
Sex (male/female)	17/6	16/8	18/6	NS
Age (years)	44.4 ± 15.1	41.9 ± 9.7	41.7 ± 8.7	NS
BMI (kg/m^2^)	27.9 ± 5.8	29.6 ± 5.8	28.3 ± 4.4	NS
Smoking (n)	11	9	7	NS
Triglycerides (mmol/l)	1.7 ± 0.9	1.6 ± 0.9	1.3 ± 0.7	NS
Total cholesterol (mmol/l)	5.4 ± 0.6	5.2 ± 0.9	5.5 ± 1.2	NS
HDL (mmol/l)	1.6 ± 0.5	1.5 ± 0.4	1.6 ± 0.4	NS
LDL (mmol/l)	3.0 ± 0.5	2.9 ± 0.9	3.2 ± 1.0	NS
Glucose (mmol/l)	5.4 ± 0.6	5.3 ± 0.4	5.1 ± 0.4	NS
Hypercholesterolemia (*n*)	4	4	5	NS
Office SBP1 (mmHg)	147 ± 17	141.6 ± 8.7	147.5 ± 14.7	NS
Office DBP1 (mmHg)	86.1 ± 10.0	87.8 ± 8.0	89.3 ± 10.5	NS
Office SBP2 (mmHg)	138.5 ± 9.1	130.5 ± 11.5	126.3 ± 11.4	0.001
Office DBP2 (mmHg)	80.9 ± 8.7	79.4 ± 9.8	79.3 ± 7.0	NS
ΔOffice SBP (mmHg)	8.9 ± 13.2	11.0 ± 10.1	21.2 ± 16.6	0.005
ΔOffice DBP (mmHg)	5.2 ± 8.2	8.4 ± 7.5	10.0 ± 10.2	NS

BMI, body mass index, HDL, high-density lipoprotein, LDL, low-density lipoprotein, SBP, systolic blood pressure, DBP, diastolic blood pressure. SBP1/DBP1, the measurements at the first visit; SBP2/DBP2, the measurements after 6 weeks. The results are expressed as mean ± SD, NS, not significant.

### Cell cultures and experimental protocol

Human umbilical vein endothelial cells (HUVECs) were sourced from the American Type Culture Collection (Rockville, MD, USA) and maintained in EBM- 2 Basal Medium with EGM-2 SingleQuots Supplements (Lonza, Walkersville, MD). During the experiments, early-passage endothelial cells (ECs) that reached ~70–80% confluency were subjected to the growth medium enriched with 20% serum from HT patients before (T0) and after treatment (T1). All experiments were performed with HUVEC cells at approximately the 10th population doubling to ensure low replicative age and reproducibility.

### Examination of endothelial cell angiogenic activity

The proliferation of ECs was examined using the Cell Proliferation Kit I (PromoKine, Heidelberg, Germany). To evaluate their migration towards a chemotactic gradient created by specific sera (20%), we utilized ChemoTx migration chambers (Neuro Probe, Gaithersburg, MD). Fluorescence measurements for both analyses were conducted using a Synergy 2 spectrofluorometer (BioTek Instruments, Winooski, VT, USA). Additionally, tube formation was monitored with the Cultrex in vitro Angiogenesis Assay Tube Formation Kit (Trevigen, Gaithersburg, MD, USA). For more detailed protocols related to EC proliferation, migration, and tubulogenesis analyses, please refer to reference [12]. In addition, we measured hypoxia-inducible factor-1 alpha (HIF-1α) levels using a Human HIF-1 alpha ELISA kit (Biorbyt, Cambridge, UK), following the manufacturer's instructions.

### Determination of cellular senescence biomarkers and effectors

The fraction of cells displaying the presence of senescence-associated β-galactosidase (SA-β-Gal) in the cytoplasm at pH 6.0 was quantified according to Dimri *et al.* [13]. In some experiments, we measured SA-β-Gal activity after exposing cells to serum from HT patients (T0), which had been preincubated with a specific TGF-β1 neutralizing antibody (400 ng/ml) for 4 h [14]. The elements of the senescence-associated DNA damage response (DDR), specifically the phosphorylated variant of histone H2A.X, known as γ-H2A.X, and the p53-binding protein 1 (53BP1), were evaluated using antiγ-H2A.X (cat #ab2893, Abcam, Cambridge, UK) and anti53BP1 antibodies (cat #NB100–304, Novus Biologicals, Abingdon, UK), as outlined in [15]. Furthermore, telomere length was quantified using the Absolute Human Telomere Length Quantification qPCR Assay Kit (ScienCell, Carlsbad, CA, USA), following the manufacturer's recommendations. The expression of senescence effector proteins, p16 and p21, was determined using immunofluorescence with rabbit antihuman p16 (#ab108349; Abcam, Cambridge, UK) and antihuman p21 (#2947, Cell Signaling Technology, Danvers, MA, USA), according to the instructions described in [16].

### Mitochondria-associated parameter analysis

Mitochondrial mass was quantified using 10-*n*-nonyl-acridine orange (NAO) fluorescence, and the inner membrane potential (ΔΨm) was determined by measuring fluorescence generated by 5,5’,6,6’-tetrachloro-1,1’,3,3’-tetraethylbenzimidazolylcarbocyanine iodide (JC-1). Both measurements were performed according to protocols described in [17].

Cytochrome c oxidase, NADH dehydrogenase, and peroxisome proliferator-activated receptor gamma coactivator-1 alpha (PGC-1α) level were quantified using kits from Wuhan EIAab Science Co., Ltd (Wuhan, China).

### Oxidative stress indices

The study investigated the generation of mitochondrial superoxides and cellular peroxides by measuring the fluorescence generated by MitoSox Red and dihydrorhodamine 123 (DHR), respectively [17]. The concentration of 8-hydroxy-2΄-deoxyguanosine (8-OH-dG) was determined using the DNA Damage (8OHdG) ELISA Kit from Biorbyt Ltd. (Cambridge, UK). Additionally, the levels of 8-isoprostane, carbonylated protein content, superoxide dismutase (SOD), and catalase (CAT) were quantified using dedicated assays from Cayman Chemical (Ann Arbor, MI, USA), according to the manufacturer's instructions.

### Serum cytokine levels

The concentration of cytokines in serum samples was quantified using appropriate DuoSet Immunoassay Development kits (R&D Systems), according to the manufacturer's instructions.

### Statistics

Statistical analysis was performed using GraphPad Prism version 9.5.1 software (GraphPad Software, San Diego, USA). We assessed the normality of continuous data distributions using the Kolmogorov–Smirnov test. For data that followed a normal distribution, we applied the paired t-test. When at least one group exhibited a non-Gaussian distribution, we used the Wilcoxon signed-rank test. When appropriate, we conducted a repeated measures analysis of variance, followed by Tukey's multiple comparisons test. A multiple linear regression analysis was performed using Statistica 13.1 software (StatSoft) on SA-β-Gal activity, changes in blood pressure (ΔOffice SBP and ΔOffice DBP), and antihypertensive drug use. Results are presented as means ± standard deviation (SD), with differences considered statistically significant at a *P*-value of <0.05.

## RESULTS

### The impact of antihypertensive drugs on endothelial cell motility

**FIGURE 1:**
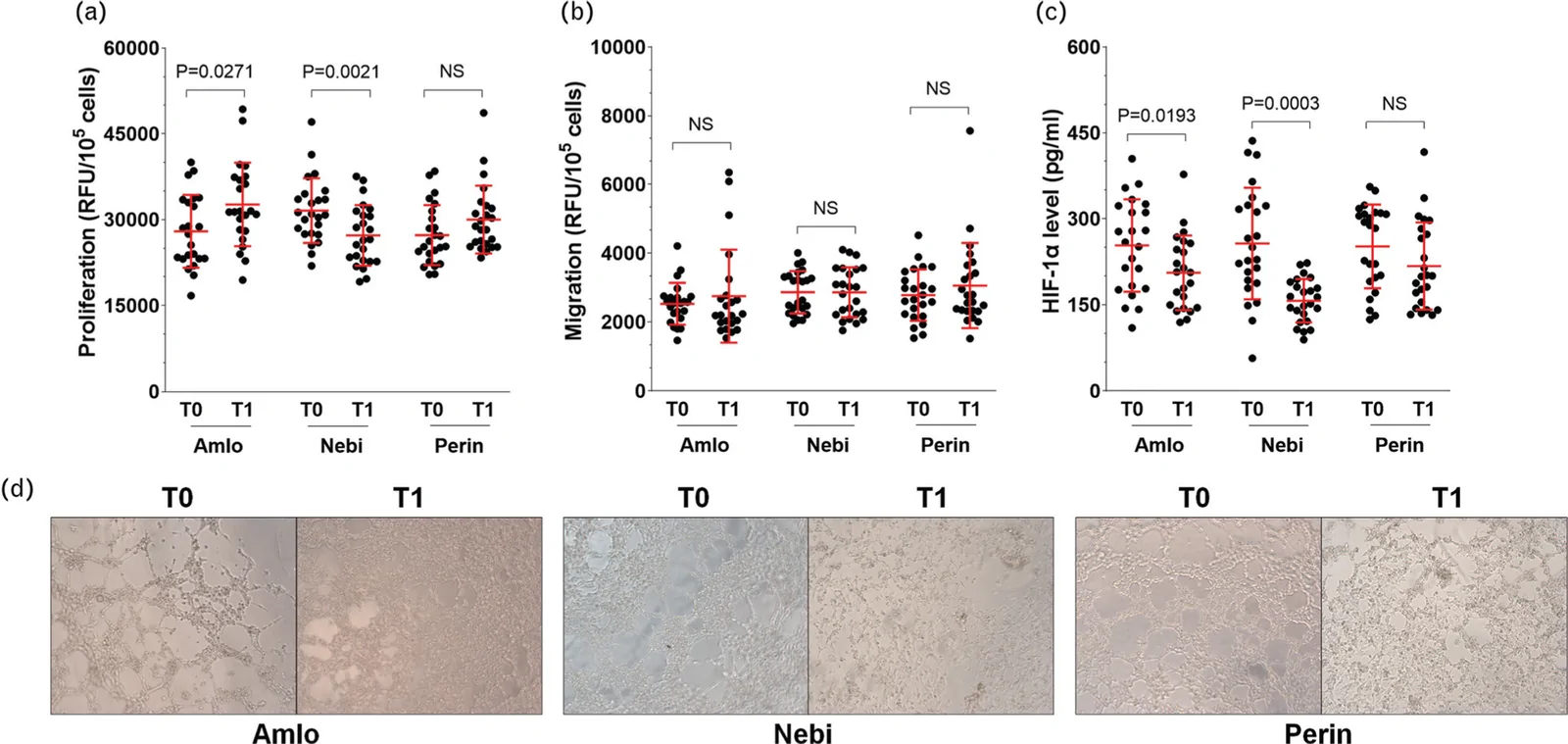
Angiogenic activity of ECs subjected to serum from hypertensive patients before and after antihypertensive therapy (a–c). T0, before treatment; T1, after treatment; Amlo, amlodipine; Nebi, nebivolol; Perin, perindopril. We visualized the ability of ECs to form tube-like structures (d). The scatter dot plots indicate the mean ± SD. Magnification ×100. NS, not significant; RFU, relative fluorescence units.

The study investigated three key parameters related to ECs’ angiogenic activity: proliferation, migration, and tubulogenesis. Additionally, the content of the transcription factor HIF-1α, which regulates angiogenic behavior in ECs, was assessed. The effects of 20% serum from patients treated with antihypertensive drugs were analyzed, with comparisons made between serum samples taken after 6 weeks of treatment (T1) and those taken before treatment (T0). The results of these experiments are shown in Fig. 1.

The results revealed that serum from patients treated with amlodipine enhanced both EC proliferation and tube formation, but notably did not influence EC migration. Surprisingly, the level of HIF-1α in these cells was down-regulated.

In contrast, serum from patients treated with nebivolol showed decreased EC proliferation and tubulogenesis. The cells lost their ability to aggregate and form characteristic chains, as illustrated in the middle panel of Fig. 1d. Similar to the amlodipine findings, the HIF-1α levels significantly decreased, while EC migration remained unchanged.

Lastly, serum from individuals treated with perindopril did not alter any of the motility parameters assessed in the study.

### The impact of antihypertensive drugs on endothelial cell senescence

**FIGURE 2:**
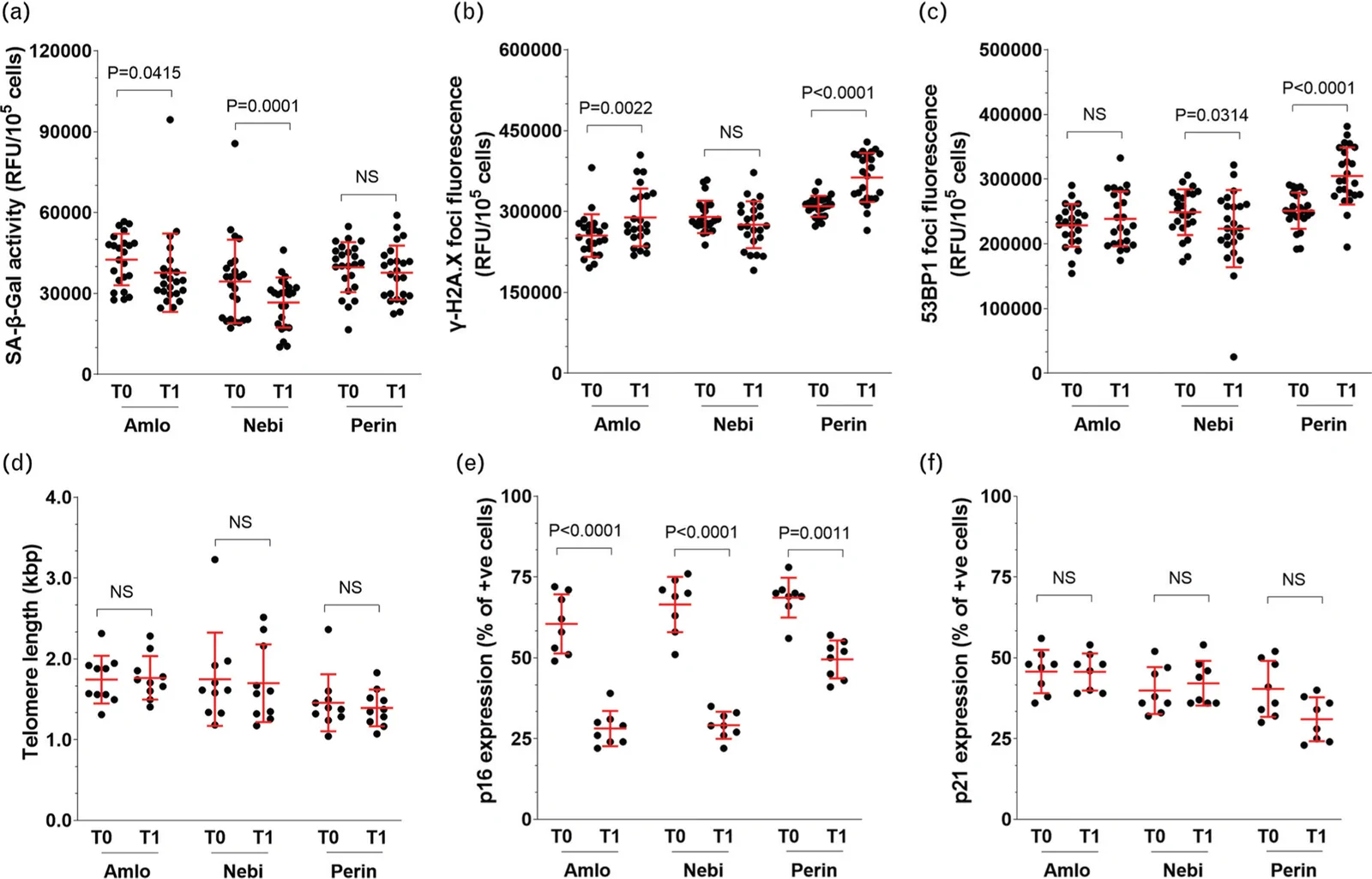
The effect of antihypertensive therapy on EC senescence. T0, before treatment; T1, after treatment; Amlo, amlodipine; Nebi, nebivolol; Perin, perindopril. The scatter dot plots indicate the mean ± SD. NS, not significant; RFU, relative fluorescence units.

Recently, we observed that indices of cellular senescence in ECs subjected to 20% serum from HT patients are more pronounced than in cells exposed to serum from healthy volunteers [9]. To determine whether antihypertensive treatment might mitigate this effect, we examined several related parameters (see Fig. 2).

Endothelial cells exposed to serum from patients treated with amlodipine exhibited a decrease in the activity of the senescence biomarker SA-β-Gal and in the levels of the cell cycle-inhibitory protein p16 (in 74% and 100% of samples, respectively). However, there was an increase in the phosphorylated form of histone H2A.X (γ-H2A.X) in 65% sera. The second component of the DNA damage response (DDR), 53BP1, remained unchanged, as did the length of telomeres and the expression of the p21 cell cycle inhibitor.

In contrast, exposure to serum from individuals treated with nebivolol resulted in reduced SA-β-Gal activity, 53BP1, and p16 levels (in 58%, 71%, and 100% of samples, respectively). The expressions of γ-H2A.X and p21, along with telomere length, did not change.

When examining serum from patients treated with perindopril, we found that SA-β-Gal activity levels were similar to those in cells exposed to serum prior to treatment. Moreover, this was observed despite a reduction in p16 levels (in 100% sera). Notably, both DDR markers were upregulated (γ-H2A.X in 96%, and 53BP1 in 92% samples), while telomere length and p21 levels remained unchanged, similar to the effects observed with the previous drug.

When the data were analyzed using a regression model, no statistically significant association was found between changes in SA-β-Gal activity and changes in SBP (*P* = 0.96) or DBP (*P* = 0.76), regardless of the antihypertensive treatment used.

### The impact of antihypertensive drugs on endothelial cell mitochondria

**FIGURE 3:**
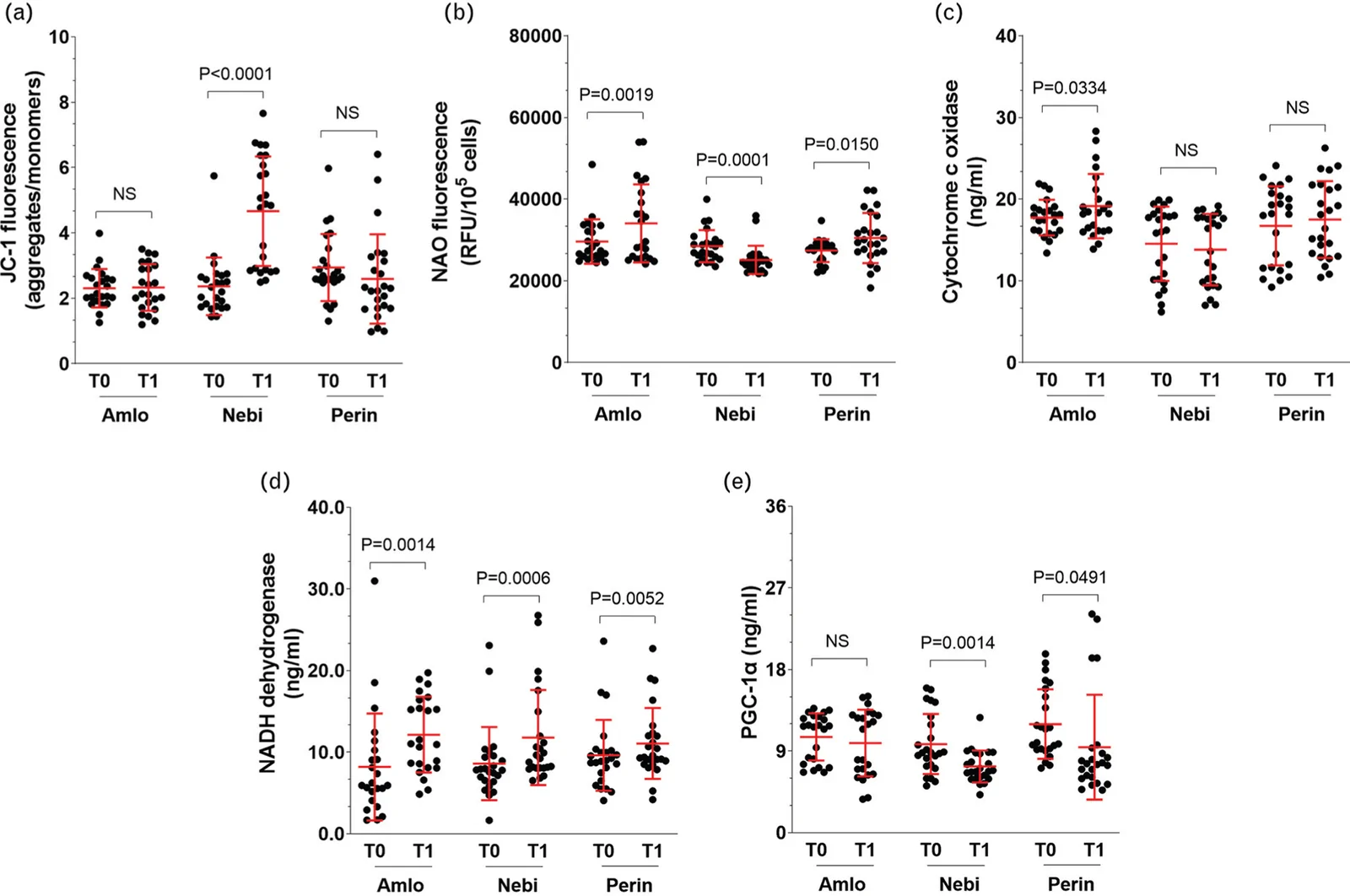
The effect of antihypertensive therapy on mitochondrial function in ECs. T0, before treatment; T1, after treatment; Amlo, amlodipine; Nebi, nebivolol; Perin, perindopril. The scatter dot plots indicate the mean ± SD. NS, not significant; RFU, relative fluorescence units.

Serum from patients treated with amlodipine exhibited increased fluorescence of NAO, indicating a higher mitochondrial mass, along with elevated activities of respiratory chain enzymes, specifically cytochrome c oxidase and NADH dehydrogenase. However, the values of JC-1 fluorescence, which reflect the inner membrane potential (ΔΨm), and the level of PGC-1α, a key player in mitochondrial biogenesis, remained unchanged (Fig. 3).

In contrast, serum from patients treated with nebivolol showed an increase in JC-1 fluorescence, indicating enhanced ΔΨm. Moreover, this was accompanied by a decrease in NAO fluorescence and a reduced level of PGC-1α. While the activity of NADH dehydrogenase was elevated, the activity of cytochrome c oxidase remained constant (Fig. 3).

In ECs subjected to serum from individuals treated with perindopril, NAO fluorescence and NADH dehydrogenase levels were elevated. However, the level of PGC-1α decreased, while the levels of both JC-1 and cytochrome c oxidase remained unchanged (Fig. 3).

### The impact of antihypertensive drugs on endothelial cell oxidative stress

**FIGURE 4:**
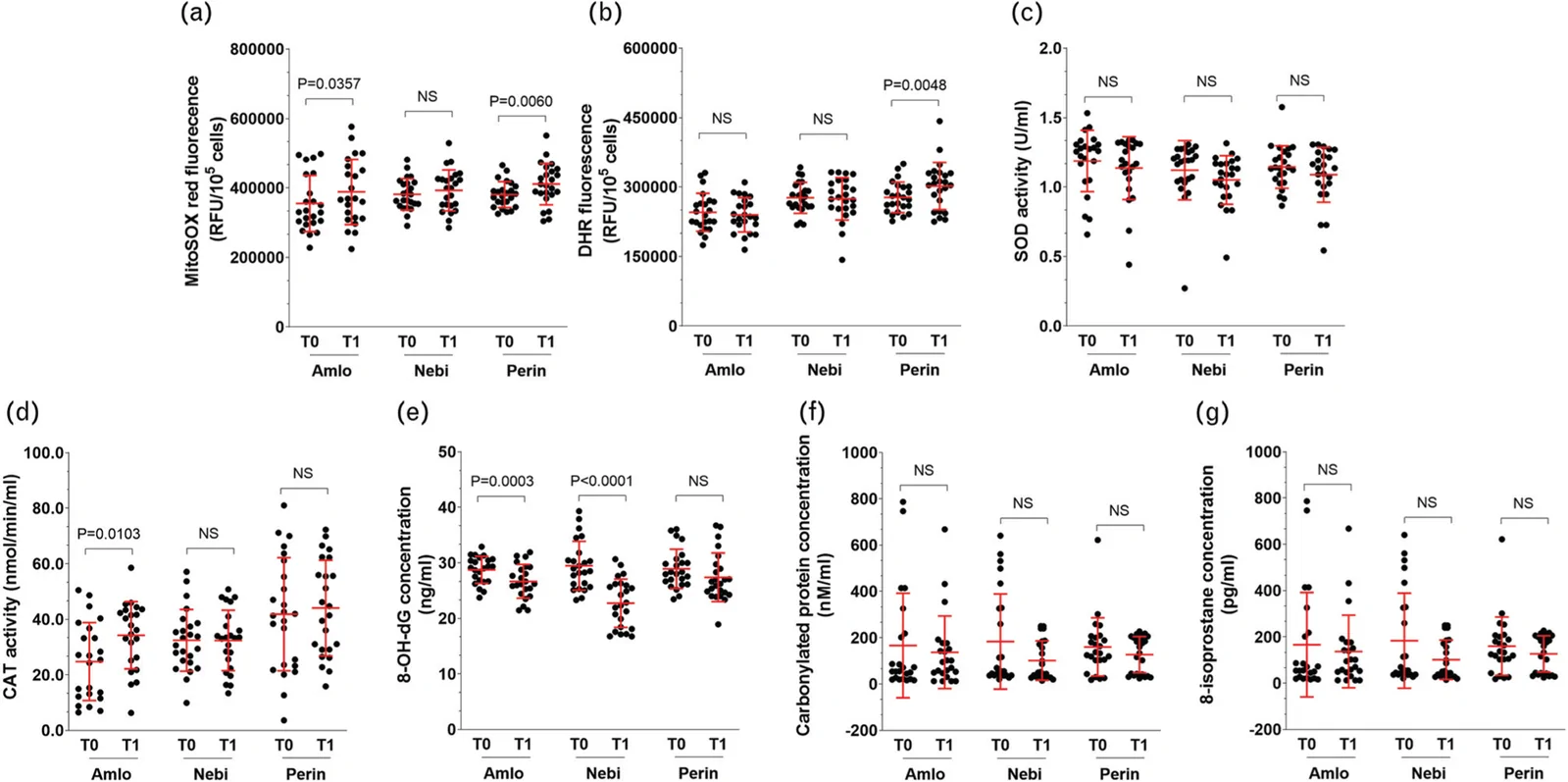
The effect of antihypertensive therapy on oxidative stress-related parameters in ECs. T0, before treatment; T1, after treatment; Amlo, amlodipine; Nebi, nebivolol; Perin, perindopril. The scatter dot plots indicate the mean ± SD. NS, not significant; RFU, relative fluorescence units.

Following the analysis of mitochondrial function, we investigated several parameters directly associated with oxidative stress. These included the production of reactive oxygen species (mitochondrial superoxides – MitoSox red fluorescence and cellular peroxides – DHR fluorescence), activities of antioxidants (SOD, CAT), and the content of oxidized macromolecules, DNA (8-OH-dG), proteins (carbonylated protein level), and lipids (8-isoprostane). The results of these investigations are depicted in Fig. 4.

ECs exposed to 20% of patients treated with amlodipine display increased MitoSOX red fluorescence and CAT activity and decreased 8-OH-dG content.

The only significant change in ECs exposed to serum from patients treated with nebivolol was a decrease in 8-OH-dG levels.

In cells treated with serum from individuals who had received perindopril, the fluorescence of MitoSOX and DHR was elevated, whereas other parameters remained unchanged.

### Changes in cytokine profile in serum from hypertension patients subjected to therapy

In the next part of the study, we quantified 14 arbitrarily selected pro-inflammatory and angiogenic cytokines associated with EC function in serum samples. The results of these measurements are shown in Table 2.

**TABLE 2:** Serum cytokine concentrations in hypertensive patients treated with amlodipine, nebivolol, and perindopril

Treatment	Amlodipine		Nebivolol		Perindopril	
cytokine	Time 0	Time 1	Time 0	Time 1	Time 0	Time 1
Angiopoietin 1 (pg/ml)	8452 ± 1368	8776 ± 1483	8716 ± 2596	7682 ± 1772^a^	9179 ± 1659	9539 ± 1152
E-selectin (pg/ml)	2779 ± 416	2816 ± 702	3012 ± 534	2101 ± 175^d^	2726 ± 414	2778 ± 340
bFGF (pg/ml)	73 ± 25	64 ± 5	77 ± 18	58 ± 10^b^	76 ± 20	57 ± 7^d^
GRO-1 (pg/ml)	438 ± 245	446 ± 272	450 ± 140	318 ± 179^b^	575 ± 380	626 ± 309
HGF (pg/ml)	634 ± 130	590 ± 193	611 ± 229	349 ± 166^d^	698 ± 136	413 ± 119^d^
IGF-1 (pg/ml)	1022 ± 262	1267 ± 462	1180 ± 162	840 ± 389^c^	1225 ± 300	1045 ± 136^b^
IL-6 (pg/ml)	105 ± 29	102 ± 44	105 ± 14	107 ± 25	111 ± 17	111 ± 16
IL-8 (pg/ml)	242 ± 112	236 ± 138	303 ± 134	291 ± 97	266 ± 49	301 ± 76^a^
MCP-1 (pg/ml)	1146 ± 389	854 ± 254^b^	1064 ± 509	622 ± 181^c^	1012 ± 414	612 ± 169^d^
P- selectin (pg/ml)	3253 ± 1115	2892 ± 1114	3326 ± 716	2625 ± 540^b^	3227 ± 999	2962 ± 1106
PDGF (pg/ml)	130 ± 52	70 ± 19^d^	121 ± 47	101 ± 23	146 ± 43	119 ± 29^c^
TGF-β1 (pg/ml)	1092 ± 227	870 ± 249^b^	1201 ± 168	801 ± 318^d^	1130 ± 121	970 ± 138^c^
TNF-α (pg/ml)	1.25 ± 0.61	2.21 ± 1.60^a^	1.93 ± 0.60	1.28 ± 0.49^b^	1.49 ± 0.57	1.58 ± 0.52
VEGF (pg/ml)	217 ± 139	177 ± 108	210 ± 124	116 ± 52^b^	201 ± 66	258 ± 152

The results are presented as mean ± SD. Statistical significance is indicated as follows:

a *P* < 0.05.

b *P* < 0.01.

c *P* < 0.001.

d *P* < 0.0001.

The cytokines of significant importance in the analyzed groups have been highlighted.

Serum samples from amlodipine-treated patients showed an increase in tumor necrosis factor α (TNFα) concentration, along with a decrease in monocyte chemoattractant protein-1 (MCP-1), PDGF, and TGF-β1 levels.

Serum from individuals treated with nebivolol showed a decreased concentration of angiopoietin 1, E-selectin, basic fibroblast growth factor (bFGF), GRO-1, HGF, insulin-like growth factor 1 (IGF-1), MCP-1, P-selectin, TGF-β1, TNFα, and vascular endothelial growth factor (VEGF).

In contrast, serum from patients treated with perindopril demonstrated increased concentrations of IL-8 alongside decreased concentrations of bFGF, HGF, IGF-1, MCP-1, PDGF, and TGF-β1.

### Comparison of SA-β-Gal activity in endothelial cells subjected to serum from HT patients upon specific treatment and TGF-β1 neutralization

**FIGURE 5:**
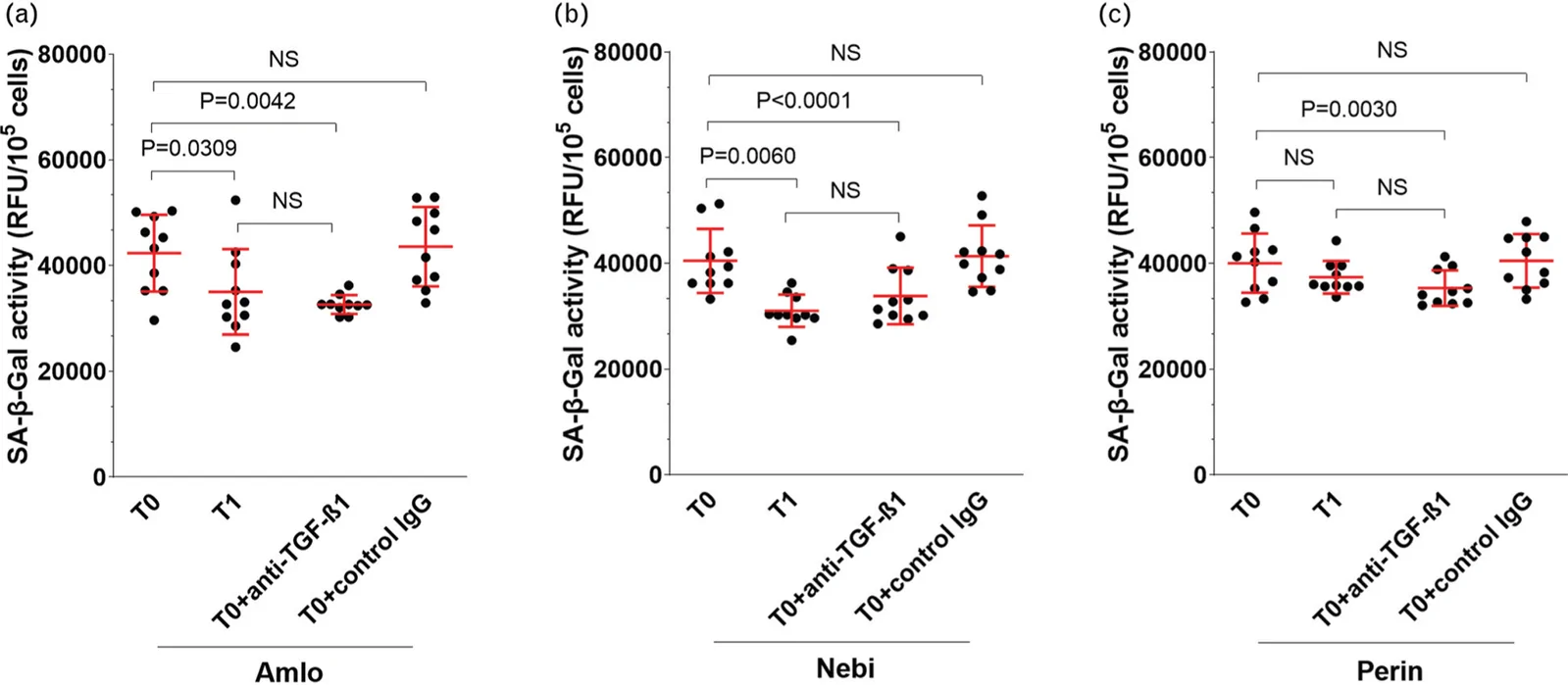
The role of TGF-β1 in inhibiting EC senescence by antihypertensive drugs. T0, before treatment; T1, after treatment; Amlo, amlodipine (a); Nebi, nebivolol (b); Perin, perindopril (c). The scatter dot plots indicate the mean ± SD. NS, not significant; RFU, relative fluorescence units.

Recently, we observed that TGF-β1 mediates EC senescence induced by serum from HT patients. Neutralizing this cytokine in serum samples reduces its pro-senescence activity [9]. In this study, we noted that all tested drugs can reduce TGF-β1 concentrations in serum (Table 2). Experiments with anti-TGF-β1 neutralizing antibodies added to serum samples taken before treatment (T0) demonstrated that this intervention reduces SA-β-Gal activity to levels comparable to those achieved by a specific antihypertensive drug, particularly amlodipine and nebivolol (Fig. 5).

## DISCUSSION

Although the bidirectional relationship between HT and EC dysfunction is well recognized and correlated with the clinical picture [2,4], the cellular and molecular mechanisms linking these two phenomena remain unclear. In this context, knowledge of the biological properties of serum from HT patients, particularly its direct effects on the vascular endothelium, is very limited. These considerations begin with the altered biochemical composition of serum in patients with HT. In fact, HT serum is clearly modified by the pathology, as evidenced by altered concentrations of various agents (e.g., galectin-3 [18], E- and P-selectins [19], interleukin-6, and TNFα [20]) that can disseminate HT-driven signals both locally and systemically. Our previous research confirmed that HT serum exhibits distinct pro-inflammatory characteristics. Additionally, it can induce premature senescence of ECs through elevated levels of TGF-β1 and intensified oxidative stress [9].

These findings open the avenue to test whether antihypertensive therapy may mitigate hypertension-related EC dysfunction and senescence. This assumption is based on a few studies that, nonetheless, show promising results.

As we have previously shown, HT serum reduces the viability of ECs [9]. This aligns with the widely accepted view that EC activity worsens as the disease progresses [2]. However, this effect is offset by increased proliferation, migration, and the ability to form tube-like structures, which are rationally aimed at preserving the integrity of the EC monolayer [9]. The effects of anti-HT drugs on these parameters varied significantly, because ECs respond differently to drugs with various mechanisms of action [18]. Specifically, serum from patients treated with amlodipine increased cell proliferation, potentiating the effect of HT, while other processes remained unchanged. In contrast, nebivolol appeared to reduce both proliferation and tubulogenesis, likely by lowering HIF-1α levels [19]. Meanwhile, serum from individuals treated with perindopril exhibited no significant effects. This finding aligns with the existing, yet inconclusive, literature regarding the effects of perindopril. For instance, one study found that rabbits treated with perindopril exhibited enhanced endothelial regrowth after arterial injury compared with those receiving a placebo [20]. Conversely, another study indicated that the active form of perindopril, perindoprilat, inhibited the formation of endothelial cell tubules, highlighting the antiangiogenic properties of this ACEi [21].

Generally, all tested parameters showed that the effects of serum from patients treated with amlodipine and perindopril are seriously inconsistent, lacking a coherent overall pattern. Amlodipine reduced the activity of the senescence biomarker SA-β-Gal. However, it also led to increased ROS production, especially mitochondrial superoxide. This elevation is likely due to the stimulation of respiratory chain enzymes, namely cytochrome c oxidase and NADH dehydrogenase [22]. The resulting increase in oxidative stress may have contributed to the rise of another senescence biomarker, γ-H2A.X, which indicates the presence of DNA double-strand breaks [23]. In order to clarify this apparent inconsistency, it is important to emphasize that SA-β-Gal is a useful, but nonspecific marker. It may not fully reflect the spectrum or timing of senescence-related changes. Parallel to this, γ-H2A.X reflects DNA damage response signaling. DNA damage may temporarily increase under conditions of oxidative stress and may occur before a stable senescence phenotype. Furthermore, increased ROS/DDR levels may cause a subset of cells to undergo apoptosis or transient growth arrest. However, since apoptosis was not directly assessed, this alternative interpretation of cell fate cannot be ruled out.

Additionally, the serum cytokine profile in this group was ambiguous. Some pro-inflammatory agents, such as TNF-αincreased, while others, such as MCP-1, decreased. The literature generally suggests that amlodipine has a direct positive effect on ECs, including increased eNOS activity and NO release [24]. It also appears to reduce nitroxidative stress in these cells [25]. However, our research indicates that, when considering the indirect, serum-dependent effects of amlodipine, the functions of ECs impaired by HT do not clearly improve.

In patients treated with perindopril, serum has been found to enhance HT-dependent EC impairment. What was indicated by increased activation of DNA damage response markers γ-H2A.X and 53BP1 in cells exposed to this serum. This uptick in DNA damage foci is likely due to elevated production of mitochondrial superoxides and cellular peroxides, which may stem from increased NADH dehydrogenase activity. These results contradict other papers showing that perindopril administration reduces oxidative stress [26]. At the same time, biochemically, the serum exhibits some signs of beneficial activity. Specifically, treatment resulted in a reduction of the inflammatory profile, as reflected by decreased levels of cytokines, including MCP-1, ICAM-1 and HGF. This observation aligns with studies demonstrating a reduction in the concentration of inflammatory markers, including CRP, IL-1α, IL-1β, and TNF-α, along with improvements in aortic distensibility in HT patients treated with perindopril [27].

Nebivolol, which effectively counteracts the HT-induced stimulation of EC angiogenic behaviors, emerged as the most promising anti-HT agent among those tested. Serum from patients treated with this drug consistently showed a beneficial effect on EC function. Decreased proliferation is likely a result of lower levels of various pro-angiogenic cytokines in the serum, specifically angiopoietin 1, bFGF, IGF-1, and VEGF. In other words, ECs receiving these signals return to a stationary nonproliferative (quiescent) state where they are ready to reinitiate divisions when required, for example, in response to mechanical damage [28]. These findings are consistent with those from studies on human coronary artery smooth muscle and ECs, where nebivolol reduced proliferative capacity in a dose- and time-dependent manner [29]. Additionally, nebivolol was found to decrease EC proliferation in ECs with increased division rates due to pulmonary hypertension [30]. A prolonged reduction in angiogenic function should be highlighted as a potential cause of increased vascular resistance and elevated blood pressure. Experiments with a longer observation period are required for a definitive interpretation of this phenomenon.

The inflammatory potential of serum modified by nebivolol treatment was reduced, as indicated by decreased concentrations of TNFα, GRO-1, HGF, MCP-1, and E- and P-selectin. Decreased levels of these cytokines may likely restore endothelium-dependent vasorelaxation. It was evidenced in obese Zucker rats, in which semapimod reduced the production of endogenous pro-inflammatory cytokines, particularly TNFα [31]. The restoration of a vasodilatory endothelium profile by nebivolol is also attributed to its ability to elevate eNOS activity and enhance NO production [32]. In this context, it is important to note that a decrease in serum TNFα also contributes to the restoration of EC NO-generating capacity [33].

Another cytokine that showed decreased levels in the serum of patients treated with nebivolol is TGF-β1. The ability of TGF-β1 to induce SA-β-Gal has been repeatedly demonstrated [34,35]. In our previous studies, we observed elevated levels of TGF-β1 in patients with hypertension. We also demonstrated that adding recombinant TGF-β1 to endothelial cell cultures for 72 h at a concentration equivalent to that found in the serum of hypertensive patients resulted in a significant increase in SA-β-Gal activity [9]. Here, we observed that ECs exposed to serum from individuals treated with nebivolol display inhibited senescence, as evidenced by decreased SA-β-Gal, 53BP1, and p16 levels. When TGF-β1 was neutralized in serum from hypertensive patients, the reduction in senescence biomarker activity closely matched that observed in cells treated with serum exposed to nebivolol. This suggests that the serum from patients treated with this drug exhibits antisenescence activity that is directly linked to the inhibition of TGF-β1 action. However, the absence of an experiment in which recombinant TGF-β1 was added to the serum of nebivolol-treated patients (to match pretreatment concentrations) limits the ability to fully explain this relationship. These findings align with experiments conducted on hypertensive Wistar rats. In this model, nebivolol attenuated HT-induced TGF-β1 elevation in the aortic media layer and normalized vascular remodeling triggered by the disease [36]. The role of nebivolol as an antisenescence agent was previously unknown; however, it is worth noting that nebivolol has been shown to increase the lifespan of long-lived mice and *Drosophila*[37].

Serum from HT patients also induces EC senescence by increasing mitochondrial oxidative stress. However, the protective effect of a spin-trap ROS scavenger can reverse this effect [9]. Nebivolol treatment effectively stabilized HT-related mitochondrial dysfunction by the changes in mitochondrial membrane potential (ΔΨm). This intervention interrupted the retrograde signaling response, which typically results in increased mitochondrial biogenesis and subsequent ROS overproduction [38]. The mass of mitochondria in ECs decreased, likely due to reduced level of PGC-1α, a key regulator of mitochondrial biogenesis [39]. Concomitant reductions in NAO fluorescence and PGC-1α protein level may reflect down-shifting of a previously activated biogenesis/compensatory program after attenuation of a stress signal[40–42]. Our findings on the effects of serum from nebivolol-treated patients on mitochondrial metabolism differ from previous observations, particularly those involving direct cell exposure to the drug. For example, other studies have shown that nebivolol hinders oxidative phosphorylation in colon and breast cancer cells [43]. In cardiomyocytes, it mitigates ANG II-induced impairment of mitochondrial biogenesis by restoring the expression of the PGC-1α gene [44]. Similar enhancements in mitochondrial biogenesis have also been seen in adipocytes treated with nebivolol [45]. Given these contradictions, we speculate that the differing effects observed in our study may result from nebivolol's indirect, serum-dependent activity on ECs. Notably, both our findings and those reporting opposite changes in mitochondrial parameters suggest that nebivolol leads to beneficial outcomes, albeit from different perspectives. Nebivolol appears to counteract the negative effects of HT, where ΔΨm values have decreased, ultimately resulting in a secondary increase in mitochondrial biogenesis [9].

Our research also showed that serum from patients treated with nebivolol stimulated NADH dehydrogenase. However, this effect was not biologically relevant, as it did not correlate with increased mitochondrial superoxide or cellular peroxide production. In fact, the level of oxidative DNA modifications, specifically 8-OH-dG, decreased, suggesting reduced oxidative stress in these cells. This decrease in DNA damage was further supported by a reduction in the expression of 53BP1, a protein that is recruited to initiate DNA repair mechanisms [46]. Decreased production of ROS and concentration of oxidized lipids, which were initially elevated by HT, was also reported in the aortic media layer of nebivolol-treated rats [36].

Overall, our findings suggest that, nebivolol was the most effective at reducing the factors associated with cellular senescence of endothelium, induced by HT. The data indicate that this benefit is mainly associated with attenuation of TGF-β1-dependent senescence, reduction in inflammatory and angiogenic signaling. Indirect evidence of changes of mitochondrial parameters was observed; however, further studies are needed to elucidate the effects of nebivolol on mitochondrial function. These observations strengthen the rationale for considering pleiotropic endothelial effects when evaluating antihypertensive therapy.

### Limitations

The study employed an in vitro serum-based model utilizing HUVEC (human umbilical vein endothelial cells). It is important to acknowledge that the findings of this study do not provide direct in vivo measurements of endothelial function and were obtained using a 20% concentration of patient serum following a 72-h exposure period, which may have influenced the observed effects. The six-week observation period for patients is a significant limitation of the study, due to the chronic nature of hypertension and the fact that the effects of the disease and the duration of treatment span many years. While the study assessed markers of cellular ageing, this did not include a direct evaluation of apoptosis. This limits the ability to interpret cellular ageing unambiguously in the group of patients taking amlodipine. The study did not assess parameters of direct mitochondrial function, such as ATP production or respiratory activity. This prevents a full assessment of the drugs’ effect on mitochondrial function, requiring further research. Additionally, the absence of an experiment involving the administration of recombinant TGF-β1 to the concentration observed in serum prior to treatment in patients treated with nebivolol does not allow for a full explanation of this association. Finally, these factors must be considered when extrapolating the results to arterial endothelial biology in hypertension.

## ACKNOWLEDGEMENTS

This research was funded by the National Science Centre, Poland, grant number 2018/02/X/NZ5/00403.

### Conflicts of interest

There are no conflicts of interest.
